# Application of chromosomal microarray to investigate genetic causes of isolated fetal growth restriction

**DOI:** 10.1186/s13039-018-0382-4

**Published:** 2018-06-04

**Authors:** Gang An, Yuan Lin, Liang Pu Xu, Hai Long Huang, Si Ping Liu, Yan Hong Yu, Fang Yang

**Affiliations:** 1 Nanfang Hospital, Southern Medical University, Guangzhou, 510515 Guangdong China; 2 Fujian Provincial Key Laboratory for Prenatal diagnosis and Birth Defect, Fujian Provincial Maternity and Children’s Hospital of Fujian Medical University, Fuzhou, Fujian China

**Keywords:** Fetal growth restriction, Prenatal diagnosis, Chromosomal microarray, Karyotype analysis, Uniparental disomy

## Abstract

**Background:**

Application of chromosomal microarray analysis (CMA) to investigate the genetic characteristics of fetal growth restriction (FGR) without ultrasonic structural anomalies at 18–32 weeks.

**Methods:**

This study includes singleton fetuses with the estimated fetal weight (EFW) using the formula of Hadlock C below the 10th percentile for gestational age. FGRs without structural anomalies were selected, and the ones at high risk of noninvasive prenatal testing for trisomy 13, 18 and 21 would be excluded. The cases were divided into two groups: early-onset group (< 24^+ 0^ weeks) and late-onset group (24–33 weeks). All patients were offered invasive prenatal testing with CMA and karyotype analysis.

**Results:**

CMA detected 10 pathogenic copy number variants and 2 variant of uncertain significance case. CMA has a 5.5% (7/127) incremental yield of pathogenic chromosomal abnormalities over karyotyping. The positive detected rate was 9.6% (5/52) in early-onset group and 9.3% (7/75) in late-onset group respectively.

**Conclusions:**

When FGR without structural anomaly is diagnosed before 33 weeks, an invasive prenatal procedure is strongly recommended. CMA can identify a 5.5% (7/127) incremental detection rate of pathogenic chromosomal abnormalities, which would impact clinical management for FGR.

## Background

Fetal growth restriction (FGR) is a common complication of pregnancy that has been associated with a variety of adverse perinatal outcomes [1]. Although many factors have been implicated in the process of fetal growth, the precise molecular and cellular mechanisms by which normal fetal growth occurs are still not well understood [2].There is a strong association between FGR and chromosome aberrations. Fetuses with chromosome disorders, including aneuploidy, duplication and deletion, are frequently growth restricted [3].

Although conventional karyotyping is the current gold standard for prenatal cytogenetic analysis for several decades, chromosomal microarray analysis (CMA) has been introduced into clinical practice, due to its high-resolution and whole-genome screening feature. Single-nucleotide polymorphism (SNP) array, a CMA platform used in prenatal diagnosis, can detect almost all genomic imbalances recognized by karyotyping, as well as smaller deletions and duplications in the kilobase (Kb) range, termed copy-number variants (CNV) [4]. It has further facilitated the detection of uniparental disomy (UPD) [5], which could also be a potential cause of FGR [6].

CMA can detect a potentially pathogenic CNV in an additional 6–7% of cases with fetal structural abnormalities detected by ultrasound [7].The American Congress of Obstetricians and Gynecologists (ACOG) and the Society for Maternal-fetal Medicine (SMFM) recommend that CMA as a first-line test is recommended when genetic analysis is performed in cases with fetal structural anomalies [8]. For those FGR fetuses without ultrasonic structural anomaly, also defined as isolated FGR, whether to implement CMA is still under consideration. In this study, we sought to investigate the genetic causes of isolate FGR by SNP array and karyotype.

## Results

A total of 155 cases with isolate FGR met the inclusion criteria. 28 cases refused to accept an invasive procedure and 127 cases were consented to participate in the study. 52 prenatal samples were obtained by amniocentesis and 75 were obtained by cordocentesis. The clinical characteristics of pregnant women included in this study were summarized in Table1.Early-onset group and late-onset one were similar regarding maternal age, height, BMI and nulliparity (Table 1).

**Table 1:** Clinical characteristics of the pregnant women

Group	Early-onset (*n* = 52)	Late-onset (*n* = 75)
maternal age (years)	33.6(19.9–43.5)	32.4(20.5–41.5)
height(cm)	160.1(154.5–171.5)	161.2(149.5–169.3)
BMI^a^	23.3(19.2-26.8)	24.1(18.5–27.4)
Nullipara	(62.4%)	(65.3%)
gestational age at diagnosis(weeks)	22.5(19.0–23.8)^b^	28.2(24.0-32.5)^b^

^a^BMI based on the weight and height at the visit of the first trimester;

^b^*P* < 0.05

Among the 127 cases, 9.4% (12/127) chromosomal abnormalities were detected totally and the clinical characteristic and related syndromes or phenotype were listed (Table 2). Taking into accounting the diagnosed gestation, the positive detected rate was 9.6% (5/52) in early-onset group and 9.3% (7/75) in late-onset group respectively. The difference between early-onset group and late-onset group is no significant (*P* = 1.00). Karyotype analysis identified 4 cases including 3 imbalanced genomes and 1 pericentric inversion. CMA detected 10 pathogenic CNV and 2 VOUS case. Compared to karyotype analysis, CMA has a 5.5% (7/127) incremental yield of pathogenic chromosomal abnormalities and a 1.6% (2/127) VOUS detected rate.

**Table 2:** Karyotype and SNP array abnormal results

Case NO.	Gestational age (weeks)	Ultrasound findings	Karyotype results	SNP array results	Length	Inheritance	Syndrome/phenotype	Pregnancy outcome
Case 1	23^+ 1^	None	45,X	arr[hg19] Xp22.33q28(168,546–155,233,731)×1	155 Mb	de novo	Turner syndrome	preterm birth
Case 2	21^+ 2^	None	46,XY	array[h19] (22)×2~3^a^	WC	de novo	Mosaic trisomy 22	In pregnancy
Case 3	23^+ 3^	None	47,XX,+mar[39]/46,XX [11]	arr[hg19] 9p24.3q13(208,454–68,216,577) × 4	68 Mb	de novo	mosaic tetrasomy 9p	TOP
Case 4	28^+ 2^	Hypoplastic Nasal Bone	46,XX,del(4)(p15)	arr[hg19] 4p16.3p15.1(68,345–35,252,743) × 1	35.1 Mb	de novo	Wolf-Hirschhorn syndrome	TOP
Case 5	22^+ 4^	None	46,XX	arr[hg19] 15q11.2(22,770,421–25,626,665) × 1	2.8 Mb	de novo	Prader-Willi syndrome	TOP
Case 6	24^+ 3^	None	46,XY	arr[hg19] 22q11.21(18,648,855–21,800,471) × 1	3.1 Mb	de novo	DiGeorge syndrome	TOP
Case 7	27^+ 6^	None	46,XY	arr[hg19] 7q11.23(72,624,203–74,143,240) × 1	1.5 MB	de novo	Willianms-Beuren syndrome	TOP
Case 8	24^+ 2^	Oligohydramnios	46,XX	arr[hg19] 14q32.33(104,856,497–107,281,980)×1,19p13.3(260,912–4,226,075)× 3	2.4 Mb 4.0 Mb	de novo	delayed development,mental retardation	TOP
Case 9	22^+ 4^	None	46,XY	arr[hg19] 3q26.33q27.2(182,374,672–185,041,523)×1	2.6 Mb	de novo	delayed development,mental retardation	TOP
Case 10	31^+ 3^	Polyhydramnios	46,XX	arr[hg19] 15q14q21.3(35,077,111–54,347,324) hmz^b^	19.2 Mb	de novo	Prader-Willi syndrome	TOP
Case 11	24^+ 3^	Tricuspid Regurgitation	46,XX, inv.(4)(p14;q28)pat	arr[hg19] 5p15.31(6,752,756–7,429,552) × 4	670 Kb	unknown	VOUS	Intrauterine death
Case 12	25^+ 4^	Echogenic intracardiac focus	46,XY	arr[hg19] 22q11.21(18,5 12,066–19,00 4772)×3	493 Kb	unknown	VOUS	Term birth

*TOP* termination of pregnancy, *WC* whole chromosome, *VOUS* variant of uncertain significance

^a^the detected sample was uncultured amniocyte

^b^a maternal UPD(15) was diagnosed by UPD tool

In Case 1-Case 10, there were ten pathogenetic CNVs de novo detected by CMA with parent-offspring analysis. In Case 1, due to an indication of isolated FGR, the patient requested a diagnosis of CMA to get more genetic information about the fetus and reduce the waiting time. CMA revealed an abnormal female chromosome complement, including the loss of one complete X chromosome. This finding is consistent with 45,X. Karyotype analysis of cultured amniocytes confirmed the CMA result. The fetus was delivered at 36^+ 4^ weeks, the birth weights of the infant were 2300 g. In Case 2, the pregnant woman experienced an amniocentesis because NIPT indicated a high risk of trisomy 22. Cultured amniocytes showed a normal 46,XY karyotype, and no trisomy 22 or small markers were observed after counting 70 metaphase cells. Interphase FISH was not selected by the patient in the prenatal testing. SNP array showed a mosaic of trisomy 22 in the uncultured samples, which was discordant with the normal result of karyotype. At present, the pregnancy is still going on. The other 8 (Case 3–10) pathogenic CNV cases were terminated with the parents’ request after the genetic counseling. Case 3 was revealed a four-copy fragment of 68 Mb in 9p24.3q13, and karyotyping demonstrated a marker chromosome with 47, XX,+mar[39]/46,XX [11]. Case 4 was shown a loss of 35.1 Mb of chromosome 4p16.3p15.1 overlapping the Wolf-Hirschhorn syndrome region. Case 5 was revealed a loss of 2.8 Mb of chromosome 15p11.2. The fetus was diagnosed as Prader-willi syndrome because of a paternal loss confirmed by trios analysis. A loss of 3.1 Mb of chromosome 22q11.21 related to DiGeorge syndrome was found in Case 6. In Case 7,CMA revealed a loss of 1.5 Mb of chromosome 7q11.23. This deletion is termed the “Willianms-Beuren syndrome”. In Case 8 and Case 9, CMA showed pathogenic CNVs related to delayed development and mental retardation according to the Decipher database. In Case 10,CMA showed a copy neutral loss of heterozygosity (LOH) of 19.2 Mb of chromosome 15q14q21.3.After trios analysis with UPD tool, a maternal UPD(15) was confirmed and the fetus was diagnosed as Prader-willi syndrome. Case 11 was confirmed a karyotype of 46, XX, inv.(4)(p14;q28),which inherited from the paternity, and CMA revealed a gain of four copies of 670 Kb in chromosome 5p15.31. No information was available for its pathogenesis. The parents refused to have further CMA trios testing. The clinical significance of this duplication is not known. Fetal death in uterus was diagnosed by ultrasound at 34 weeks. In Case 12, SNP array revealed a gain of 493 Kb of chromosome 22q11.21. This segment highly varied according to the database. The origin of the gained copy was unclear due to the parents’ decline. A male infant was delivered with a 2300 g birth weight at 37^+ 4^ weeks and following up to 10 months was normal. The rest cases with negative results had no identifiable phenotype at birth.

## Discussion

Several organizations recommend that CMA should be applied to detect genetic abnormalities in fetus with structural anomalies. Significant fetal growth restriction is often seen with trisomy 13 and trisomy 18, which are usually confirmed multiple malformations by ultrasound. But, it is ambiguous that whether a pregnant woman should accepted the invasive prenatal procedures when a FGR is diagnosed with a normal ultrasound scanning. In this study, we determined the incidence and patterns of chromosomal abnormalities in a cohort of 127 FGR without structural anomalies. The overall detection rate of chromosomal abnormalities were 9.4% (12/127).We explored the genetic abnormalities of FGR diagnosed at different gestational ages, However, there was no statistical difference between the early-onset group and late-onset one (9.6% vs. 9.3%, *P* = 1.00).The reason may due to limited sample size. We still recommended that it was reasonable to discuss the probability of an invasive prenatal procedure with pregnant woman in case of isolate FGR diagnosed before 33 weeks. Although Merel recommends that testing for chromosomal anomalies should be offered in case of FGR between 18 and 24 weeks gestation [9].

Frequently, FGR is a major and only manifestation in the prenatal diagnosis of some micro-duplication/deletion syndromes, and intellect disability or delayed development was solely clinical presentation after birth. Due to the limited resolution of karyotype analysis, many well-characterized disease-causing genetic variants could not be detected. This study demonstrated 5 pathogenic CNV de novo (Case5–9), which only detected by CMA, because the genetically material imbalance was less than 5 Mb in length. After genetic diagnosis, each case was re-evaluated by ultrasound. Just like in Case 6, DiGeorge syndrome was found by CMA in the isolated FGR fetus and confirmed by FISH. Common ultrasound anomalies included palatal anomalies, cardiovascular anomalies and scoliosis [10] were easy to confirm, however, this case was just an isolated FGR without any structural anomaly. At 27 weeks, a smaller thymus gland was confirmed by Ultrasound before termination of pregnancy with the parent’s request.

Uniparental disomy (UPD) is another genetic cause of FGR. The concept of UPD was first introduced by Eric Engel, owing to the fact that both members of such a pair of chromosomes from only one parent [11]. The pathogenesis of UPD is determined by both epigenetic imprinting as well as demasking of autosomal-recessive diseases (homozygosity by isodisomy). It is striking that many UPDs are associated with disturbed intrauterine growth [12]. A maternal uniparental disomy (mUPD) in which two copies of chromosome 15 of maternal origin accounts for 20–25% of Prader-Willi syndrome (PWS) [13]. A study from the UK supports that the changing genetic subtype proportions of PWS are due to an increase in the numbers of mUPD babies because of the increasing proportions of older mothers [14]. The incidence of UPD is estimated to be approximately 1:3500 live births and growing numbers of patients will be detected with the advent of the whole-genome techniques [15]. As in Case 10, a 41-year-old woman accepted cordocentesis procedure with Indication of isolated FGR and decreased fetal movement at 31^+ 3^ weeks of gestation. Finally, a UPD(15)mat has been confirmed by trios analysis with UPD tool [16], and the fetus was diagnosed with PWS. So UPD should deserve more attention in FGR cases especially with advanced-aged pregnant women.

In the prenatal setting, it may be difficult to interpret the significance of a CNV due to the limitations of fetal imaging and the limited information currently available correlating prenatal CNV findings with postnatal phenotype [17]. VOUS may cause considerable stress and anxiety as the parents may not obtain a satisfied expectation. The parents refused to have further testing. Case 11 exhibited a chromosome complement with the gain of 670 Kb in chromosome 5p15.31 involved two genes: PAPD7 and ADCY2. Four copies were detected and the breakpoints occurred in PAPD7 and ADCY2 respectively, revealed by CMA. So far, both of the genes whether cause disorders has not been reported. Parental DNA was not available for further analysis and the fetus died in uterus at 34 weeks. We were unable to determine whether the duplication had occurred de novo. Similarly, a VOUS was defined in Case 12. Finally, the VOUS detected rate was 1.6%, similar to the literature [18].

CMA does not provide information about the chromosomal mechanism of a genetic imbalance. For example, the fetus of Case 8 was diagnosed as mosaic tetrasomy 9p with the combination of CMA and karyotype analysis. The components of the prenatal sample would change following the culture process, which increased the uncertainty of diagnosis results. In Case 2, the result of CMA indicated a mosaic of trisomy 22 and the karyotype was normal. Maybe, the direct detection of uncultured samples by CMA can more fully represent the genetic characteristics of the fetus.The ACOG and SMFM recommend that providers discuss the benefits and limitations of CMA and conventional karyotype with patients, and that both options are available to women who choose to undergo prenatal diagnostic testing [19]. QF-PCR or FISH analysis would be an alternative in the rapid prenatal test for trisomy 13, 18, 21and sex chromosome aneuploidies. But CMA could detect micro-deletion/duplication with a high-resolution view of the whole genome and copy neutral LOH with platforms incorporating SNP probes. More pregnant women are willing to choose CMA in the prenatal setting.

Inevitably, a limitation of the prospective study is that the postnatal follow-up is still pending, in order to acquire the long-term growth and development aspects of born cases. How to select a proper criterion to define FGR is another puzzle [20]. The ultrasound limit for FGR is fetal weight (EFW) or abdominal circumference (AC) under the 3rd, 5th, 10th percentile or below 2 Standard Deviation (SD) from the population standard or reference [21]. We choose the 10th percentile as a cut-off to avoid omitting the so-called “mildly growth restricted” which are at increased risk for complications between the 3th and 10th percentile [22]. This fact may result in a selection bias leading to underestimated incremental yield of CMA.

## Conclusion

In summary, as underlying factors in case of FGR, aneuploidy, submicroscopic abnormality and UPD should be considered comprehensively. An invasive prenatal procedure is strongly recommended when FGR is diagnosed before 33 weeks. CMA detected all the chromosomal aberration detected by karyotyping, and has a 5.5% (7/127) incremental detection rate of genetic cause of isolated FGR, which could impact the clinical decision. CMA as the first-line test plus karyotyping is effective and feasible as a joined prenatal testing for suspected FGR cases.

## Methods

### Ethics and cases selection

The study was approved by the ethics review boards of Nanfang Hospital (No.NFEC-2016-093) and Fujian Provincial Maternity and Children’s Hospital (No.12). Written informed consent was obtained in all cases.

This prospective multi-centers cohort study consisted of singleton FGR cases underwent invasive prenatal diagnostic testing at Fujian Provincial Maternity and Children’s Hospital (FPMCH) and Nanfang Hospital (NFH) of Southern Medical University from July 2015 to February 2018. These two hospitals are tertiary referral prenatal diagnosis center in each province. Gestational age (GA) was assessed according to last menstrual period (LMP) and crown-rump length (CRL) at 11 to 13^+ 6^ weeks. Inclusion criteria was FGR without structural anomalies diagnosed by ultrasound when the EFW is less than the 10th percentile for gestational age based on the formula of Hadlock C [23]. At high risk of noninvasive prenatal testing for trisomy 13, 18 and 21, multiple gestation, chronic nephropathy, preeclampsia, antiphospholipid syndrome, TORCH infection, substance use and abuse were excluded. All the morphology scan were performed by experienced operators according to the practice guidelines for performance of the routine fetal ultrasonic scan released by the International Society of Ultrasound in Obstetrics and Gynecology(ISUOG). Based on the gestational age at diagnosis, the cases were divided into two groups: early-onset group (< 24^+ 0^ weeks) and late-onset group (24–33 weeks). All women received pre-test counseling regarding the procedure-related risks and benefits from karyotype and microarray. Women with positive results were offered fully counseling by the fetal medicine specialists. All the prenatal samples obtained by amniocentesis or cordocentesis were processed in parallel using both SNP array and G-banding for conventional karyotyping.

### Karyotype analysis

Amniotic Fluid or fetal cord blood samples were obtained according to the prenatal procedure protocol [24]. The cultured amniocytes or lymphocytes were analyzed by routine cytogenetic analysis using G-banding techniques at a resolution of 400–500 bands. The number of cells examined varied between 20 and 30.The results of cultured amniocytes are available within 14 to 20 days and the ones of lymphocytes within 5 to 7 days.

### SNP array and data interpretation

A CytoScan 750 K array (Affymetrix Inc., Santa Clara, CA, USA) was used for assessing the prenatal samples, which covered over 750,000 markers distributed across the entire human genome, including 200,000 probes for single nucleotide polymorphisms (SNPs) and 550,000 probes for copy number variations (CNVs). Hybridization, data extraction and analysis were performed as per the manufacturer’s protocol. A resolution was generally applied: gains or losses ≥400 kb and loss of heterozygosity (LOH) ≥ 10 Mb [25]. The results were analyzed with Chromosome Analysis Suite (ChAS) software (Affymetrix, USA), using annotations of the genome version GRCH37 (hg19). The Database of Genomic Variants (DGV), the Database of Chromosome Imbalance and Phenotype in Humans Using Ensemble Resources (DECIPHER), the International Standards for Cytogenomic Arrays Consortium (ISCA), OMIM genes and the local database were used to evaluate the CNVs identified in this study. The CNVs were classified as benign, pathogenic, or variants of uncertain significance(VOUS) according to the American College of Medical Genetics (ACMG) guideline [26]. Blood samples were collected from both parents and were analyzed if variants of uncertain significance (VOUS) were detected in the fetal sample by CMA.

### Statistical analysis

SPSS software v20.0 (SPSS Inc., Chicago, IL, USA) was used for statistical analysis of the data. Statistical comparisons were performed using the chi-square test and Fisher exact test was used in cases where a table cell contained < 5 observations. Differences were considered as statistically when *P* < 0.05.
